# Exploring Teacher Job Satisfaction in Rural China: Prevalence and Correlates

**DOI:** 10.3390/ijerph19063537

**Published:** 2022-03-16

**Authors:** Huan Wang, Claire Cousineau, Bill Wang, Lucy Zeng, Andrew Sun, Ezra Kohrman, Nick Li, Esther Tok, Matthew Boswell, Scott Rozelle

**Affiliations:** Stanford Center on China’s Economy and Institutions, Stanford University, 616 Serra Mall E501, Encina Hall, Stanford, CA 94305, USA; clairece@stanford.edu (C.C.); bwang20@thehill.org (B.W.); lucyzeng@stanford.edu (L.Z.); andrewsun@uchicago.edu (A.S.); ekohrman@stanford.edu (E.K.); axnli@ucdavis.edu (N.L.); etok@stanford.edu (E.T.); kefka@stanford.edu (M.B.); rozelle@stanford.edu (S.R.)

**Keywords:** education, teacher job satisfaction, rural China

## Abstract

Extant research continues to establish the importance of teacher job satisfaction to student performance, yet teacher job satisfaction remains under-investigated in rural China. In this paper, we examine the prevalence and correlates of teacher job satisfaction. Using data from 634 teachers across 120 schools in rural China, we find an alarmingly high prevalence of teacher job dissatisfaction: roughly 21% of rural teachers were less than satisfied with their jobs. In addition, we find that several individual- and school-level characteristics, including being a male teacher, being a homeroom teacher, not having a management role in school, being a middle-aged teacher, and a school’s boarding status, are correlated with teacher job dissatisfaction. In sum, the results demonstrate a need for further research and policy interventions to improve teacher job satisfaction in rural schools.

## 1. Introduction

In recent years, China’s education system has improved with China’s economic growth. The adult literacy rate’s increase from 66% to 97% between 1982 and 2019 reflects this trend [1]. Students from China’s large cities have achieved some of the highest scores on international standardized evaluations, such as the Organization for Economic Cooperation and Development (OECD)’s Program for International Student Assessment (PISA) [2]. Although, by such measures, China’s education system appears to have improved significantly, large disparities remain between students’ academic performances in rural and urban China. As early as primary school, children in rural China fall behind those in urban areas across a wide spectrum of educational outcomes [3]. Research has shown that this gap exists not only at the national level, but also within provinces [4]. These performance disparities have also led to significant differences in educational attainment between students in urban and rural areas [5,6,7].

Because rural children make up approximately 70% of China’s school-aged population [8], this achievement gap may have a negative impact on China’s economic future. Without narrowing the urban-rural achievement gap, it is possible that rural students will not be able to achieve the skills necessary for high-wage jobs that power a developing nation’s transition to an advanced, high-income economy [9,10]. A large, under-skilled fraction of the population could serve as a drag on growth and increase the risk of economic stagnation typical of the “middle-income trap” [10,11,12].

Previous research has found many reasons behind why rural students in China struggle in school, including dilapidated school facilities, a lack of resources, and poverty [8,13,14,15]. However, a less studied factor that may drive low school performance among rural children is low teacher job satisfaction. Research has found that student academic performance is correlated with their teachers’ level of job satisfaction. For instance, one study found that students of more satisfied teachers perform better in both reading and math [16]. Additional evidence points to the importance of teacher enthusiasm in student outcomes, from interest in learning to student recall; teacher job satisfaction is one of the key contributors to an enthusiastic teacher [17]. Finally, teacher job satisfaction and teacher self-efficacy have been found to be positively and strongly correlated, and teachers with higher rates of self-efficacy tend to produce better student outcomes [18,19]. On the other hand, low teacher satisfaction has a significantly negative correlation on students’ academic performances: Liu and Chen found evidence suggesting that teachers’ job dissatisfaction is closely associated with teacher absenteeism and higher rates of attrition from the teaching profession [20,21].

Extant literature tends to disagree on how certain individual-level characteristics, such as gender and age, are associated with higher levels of teacher job satisfaction. For example, several studies have found that female teachers are typically more satisfied than their male counterparts [22,23]. Other studies, however either do not find any correlation between job satisfaction and gender or find female teachers to be more stressed and less satisfied [19,24,25,26]. Similarly, while one study found that younger teachers are more satisfied than their older counterparts [24], another study finds that with more years of teaching experience, teachers become increasingly satisfied [19]. Moreover, one study also found that less educated teachers tend to be more satisfied than their more highly educated counterparts [24]. Certain environmental characteristics have also been found to support satisfaction levels. Teachers in areas with high levels of social support and internal school cooperation have been more satisfied, while teachers with heavier workloads have been found to be less satisfied than those who perceive their workload as manageable [19,22,24]. In sum, there seems to be little agreement on the general characteristics associated with teacher satisfaction, which suggests the importance of context-specific research.

While previous research has gone some way in exploring the characteristics of satisfied teachers and highlighting a link between teacher job satisfaction and student performance, several gaps in understanding remain. First, the most recent highly cited paper on teacher job satisfaction in rural China was published in 2005. Since then, China’s economy has grown remarkably, from $2.3 trillion in 2005 to $12.3 trillion in 2017 [27]. In addition, the central government has made major investments and changes to China’s education infrastructure. Since 2013, it spent 15.7 billion RMB to incentivize young teachers to work in poor and rural areas. In addition, China’s local governments began the School Mapping Restructure (SMR) program, causing many small schools to close or merge with larger primary schools [28]. Moreover, other projects have continued to improve rural schools’ classroom environment by equipping rural classrooms with computers for more than 31 million students between 2002 and 2007 [29]. These changes all may have significantly impacted teacher experience due to their large scale, and yet, we have little understanding about whether these investments have impacted teacher job satisfaction.

Second, existing literature lacks international context, which is often due to the lack of a standardized measure. Past research has compared factors that influence teacher job satisfaction in China to those described in the international literature [30,31], but do not measure teacher job satisfaction using an international scale. Without internationally standardized scales, it is challenging to accurately understand teacher job satisfaction levels in regions across China when compared to the rest of the world.

The purpose of our study is to address these gaps by providing a more comprehensive picture of teacher job satisfaction in rural China. To achieve this goal, we pursue two specific objectives. First, we document the prevalence of job satisfaction among teachers in our sample of rural China and compare this to other countries using an international comparative metric. Second, we identify the teacher and school characteristics that are correlated with teacher job satisfaction in the rural context. The rest of the paper is organized as follows. Section 2 introduces sampling methods, data collection, and methods for measuring teacher job satisfaction in rural China. Section 3 reports the prevalence and correlates of teacher job satisfaction. Section 4 discusses the results. Section 5 concludes.

## 2. Materials and Methods

### 2.1. Sampling Procedure and Data Collection

The study was conducted according to the guidelines of the Declaration of Helsinki and approved by the Institutional Review Board (or Ethics Committee) of Stanford University (Protocol ID 32594). 

We conducted our survey in three counties in the southern part of Jiangxi province in China. All three counties are nationally designated poor counties identified by the Chinese government in 2012 as areas with extreme poverty. The indicators used to identify poor counties include per capita GDP, per capita general budgetary revenue, and rural per capita net income [32]. The economic development in the three counties is lagging behind the average of China as well as of other areas in Jiangxi province. Per capita GDP in the three counties was less than 3200 USD in 2015, which is around 40% of the national average [7,33]. In addition, more than 80% of the population are rural residents, in comparison to 44% across China and 48% across Jiangxi province [7,33]. 

To select our sample, we followed a two-step sample selection protocol. The first step of our research design involved selecting a representative sample of schools from the three counties. We used official records from county education bureaus to create a population frame of all rural, public primary schools in the three counties. According to the records, there was a total of 458 schools. In each of the townships, we randomly selected schools using a sampling fraction that is proportional to that of the total number of schools. Finally, we randomly selected 120 schools. Of these, 37 schools (30.8%) were in County A, 25 schools (20.8%) were in County B, and 58 schools (48.3%) were in County C. In this way, our sample is representative of the three counties being studied.

After selecting schools, we sampled classes. We conducted our study among the fourth and fifth grades of each of the sample schools. We then randomly selected at most two classes in each grade in each school and surveyed all Chinese and Math teachers in the sampled classes. Ultimately, we surveyed a total of 634 teachers in 288 classes in these 120 schools.

### 2.2. Outcome Measures

The Progress in International Reading Literacy Study (PIRLS) Teacher Job Satisfaction Scale (TJS) is used to produce an internationally recognized quantitative measure of the level of each teacher’s job satisfaction levels.

The TJS scale is an international comparative assessment of teacher job satisfaction levels developed by the Trends in International Mathematics and Science Study (TIMSS) and Progress in Reading and Literacy Study (PIRLS) assessment [34,35,36,37,38]. This scale has been used in 52 countries and regions representing a variety of development and income levels. The survey was translated into Mandarin Chinese and the translation was verified according to the PIRLS translation guidelines [39]. The TJS scale has been used in Chinese context as reported in the PIRLS assessment. The TJS scale also has good reliability among teachers in rural China with a Cronbach’s alpha reliability coefficient of 0.923. The Cronbach’s alpha reliability coefficient for our sample is 0.915.

The TJS scale also has good construct validity. It consists of five items related to job satisfaction. In the case of each of these items, the participants rated how often they had certain feelings about the listed items. The five items on the scale were: (a) I am content with my profession as a teacher; (b) I find my work full of meaning and purpose; (c) I am enthusiastic about my job; (d) My work inspires me; and (e) I am proud of the work I do.

To create a raw score for the TJS scale, each response was assigned a numerical value (“very often” = 2, “often or sometimes” = 1, and “never” = 0). The raw scores range from 0 (never feel satisfied with any of the five items) to 10 (satisfied with all of the five items). A lower TJS score therefore corresponds to a lower level of job satisfaction. Following PIRLS protocol, raw scores were transformed into TJS transformed scale scores, which allows us to compare the TJS scores of our sample teachers with teacher TJS scales from a large set of international surveys/studies [36]. 

In the second part of the survey, we collected individual teacher characteristics, such as each teacher’s gender, age, teaching experience, working hours, job rank, and education degree. We also collected data on whether the teacher is a homeroom teacher, whether the teacher has a school administrative role, the distance from the school to the county seat, and whether there are boarding students in school.

### 2.3. Data Analysis

Our quantitative analysis is comprised of two parts. To address our first objective, we use descriptive analyses to look at the prevalence of teacher job satisfaction in rural China and compare it to other countries and regions. To address our second objective, we compare the TJS scores between different teacher and school characteristics to understand what kinds of teachers are more likely to have higher or lower job satisfaction levels. We use t-tests to measure if there is a significant difference in teacher job satisfaction level between two categories, such as teacher gender or whether the teacher is a homeroom teacher. We also use an F-test to examine joint significance among different groups when there are more than two categories, such as teacher age groups, teacher rank, and education degree, as well as school distance to the county seat and school size.

## 3. Results

### 3.1. Summary Statistics

Table 1 presents the socioeconomic and demographic characteristics of the study participants. Of the sampled teachers, about half were female teachers. The average teachers’ age was 34 years old, though ages ranged from 20 to 63 years. About one third of the teachers in our sample were above 35 years, which indicates that the majority (66%) of teachers were younger than 35. Most teachers (80%) had taught for more than five years, and 23% of them had more than 20 years of teaching experience. About 60% of the teachers in our sample were homeroom teachers. Although all the teachers in our sample were math or Chinese teaches, about 12% of them were also principals, meaning they held a major administration role in their school. With respect to working hours, 95% of teachers reported working over 8 h per day, while 87% of teachers reported having to work weekends. Additionally, roughly one third of teachers indicated that they helped students with work outside of class. Forty-six percent of teachers had a vocational high school degree, 5% had a high school pedigree, 42% had a vocational college degree, and less than 7% held a bachelor’s degree; this indicates that roughly 93% rural teachers never went to college. Most teachers, out of a 4-tier ranking system, had the second and third highest credential ranking.

Table 2 presents the summary statistics of the sampled schools. The average number of students per school was roughly 486, with 48 students boarding per school. Every teacher was, on average, responsible for 18 boarding students. The average distance from the school to the county seat was 30.43 km, while the average distance from each school to the farthest village was roughly 7 km.

### 3.2. Prevalence of Teacher Job Dissatisfaction

Our results show that the job satisfaction level among our sample’s rural primary school teachers in China is low (Table 3). Of the sampled teachers, approximately 21% were less than satisfied with their job. Moreover, only about 28% of the rural teachers in our sample are “very satisfied” with their profession. Finally, on the scale of 0 to 10 TJS, our sample’s raw average TJS scale score was 4.6. Following PIRLS protocol, raw scores were transformed into TJS scaled scores. The transformed score was 7.95.

When examining teacher responses to each of the five statements about being a teacher in the TJS scale, we find that teachers felt less satisfied in certain statements than others (Table 3). Of our sample teachers, over 45% never or almost never felt that they were content with their profession as a teacher. The percentage of teachers never or almost never finding their work full of meaning and purpose was about 22%, which is the same as the percentage of teachers never or almost never feeling enthusiastic about their job. More than a quarter of the rural teachers (29%) never or almost never felt that their work inspired them. About 35% never or almost never felt proud of the work they do as a teacher.

**Figure 1:**
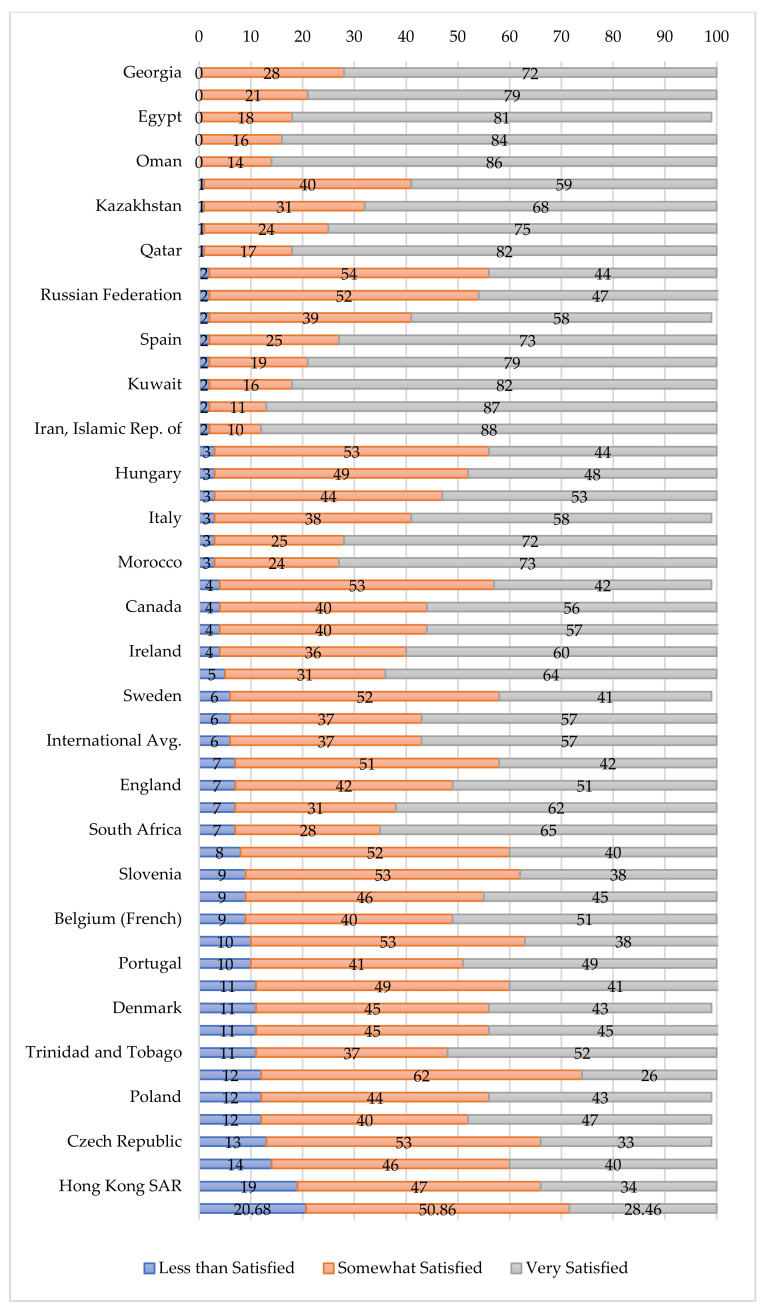
Comparison of prevalence of teacher job satisfaction levels between samples from rural China and other countries/regions. Note. Teacher job satisfaction levels are categorized as “Very satisfied,” “Somewhat satisfied,” and “Less than satisfied” according to the cutoffs used in TIMSS & PIRLS surveys. Data source: Progress in Reading and Literacy Study and author’s survey.

**Figure 2:**
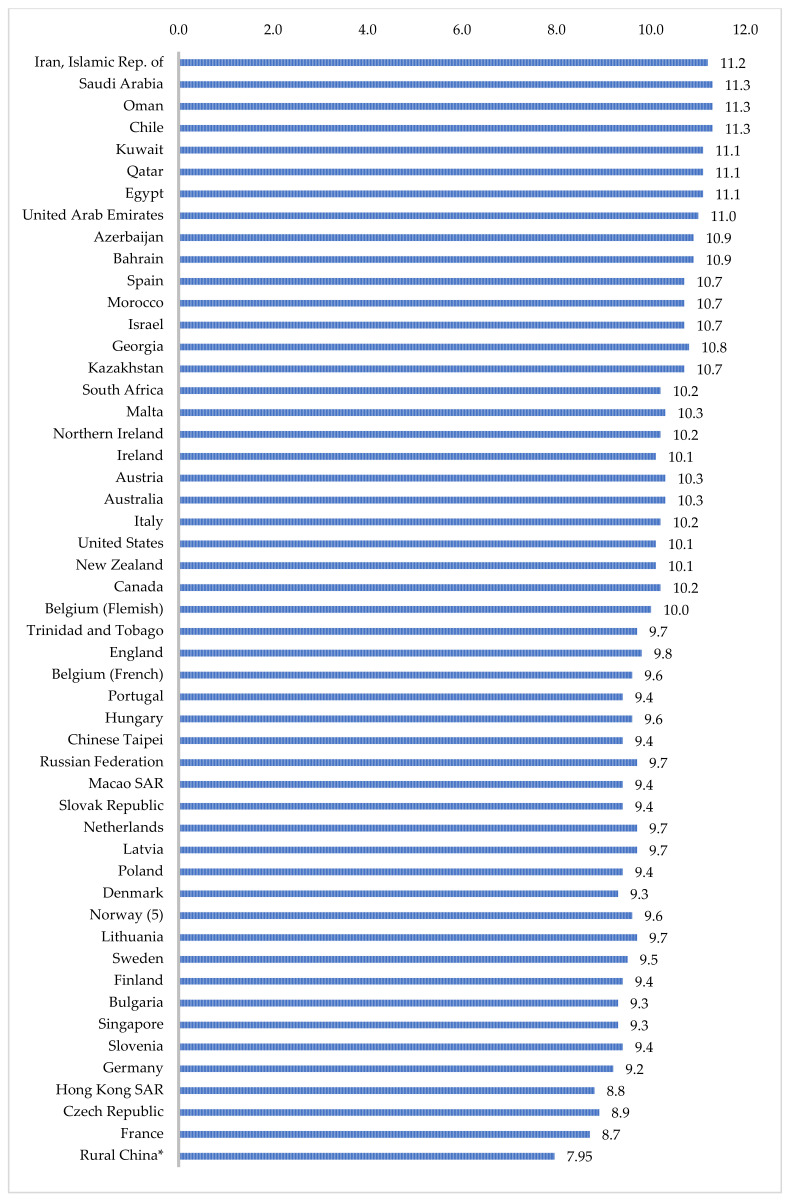
Comparison of average teacher job satisfaction (TJS) scale scores between samples from rural China and other countries/regions. Note: To enable comparisons across countries and regions, TJS raw scores have been converted to transformed scores according to the PIRLS conversion chart. Data source: Progress in Reading and Literacy Study and author’s survey.

Additionally, of the sampled teachers, approximately 21% were less than satisfied with their job, a number almost three times higher than other countries and regions across 52 countries reported by the same TIMSS & PIRLS survey (Figure 1). Across countries that participated in the PIRLS, only 6% of teachers were categorized as less than satisfied teachers. Moreover, only about 21% of the rural teachers in our sample were “very satisfied” with their profession. This is much lower than the 57% PIRLS report in other countries. In addition, our sample’s average TJS scale transformed score was 7.95, which puts our sample region at the lowest rank of 52 countries or regions (Figure 2). 

### 3.3. Correlates of Teacher Job Dissatisfaction

In Table 4, we present factors that are significantly correlated with teacher satisfaction levels. Several individual-level factors were significantly related to the teacher job satisfaction scores. Male teachers had lower teacher job satisfaction scores than female teachers (4.44 for male teacher compared to 4.97 for female teacher, significant at the 5% level). Age also had a statistically significant correlation to the teacher job satisfaction scores. For example, teachers above the age of 46 had the highest average job satisfaction score of 5.236, while teachers below 25 had the second-highest average job satisfaction score of 5.026. Teachers between the ages of 26 and 35 had the lowest score of 4.418, while teachers in the group between 36 and 45 years old scored 4.602, on average. The differences in job satisfaction scores among these age groups are significant at the 5% level.

Evidence also indicates that a teacher’s role in school appears to be related to their levels of satisfaction. Homeroom teachers tended to have a significantly lower teacher job satisfaction score than non-homeroom teachers, with an average score of 4.429 compared with 5.016. This difference is significant at the 5% level. Additionally, having a school management title also was a predictor for higher teacher job satisfaction; those with a management title had an average job satisfaction score of 5.912, while those without had an average score of 4.483. This difference is significant at the 1% level.

However, other teacher-specific factors were not found to be statistically related to teacher job satisfaction scores. For example, teacher rank and certification status were not significantly correlated with teachers’ job satisfaction scores. A teacher’s education degree, too, did not have a significant relationship with a teacher’s job satisfaction score.

School characteristics, such as distance from the county seat and school size, were not found to be a significant predictor of teacher job satisfaction scores. However, teachers who worked at boarding schools exhibited lower levels of satisfaction than teachers who did not work at boarding schools (*p* < 0.10). While teachers at schools without boarding students had the average job satisfaction score of 4.480, those at schools with boarding students had the score of 4.758. This difference is significant at the 10% level.

In review, gender, homeroom teacher status, leadership status, age, and boarding status were significant predictors of teacher job satisfaction. Several other variables, however, were not correlated with levels of teacher job satisfaction, including certification status, credential status, the number of degrees held by teachers, the school distance to the county seat, and school size.

## 4. Discussion

This paper is one of the first to provide an updated estimate of the prevalence and correlates of teacher job satisfaction in our sample of rural China using an internationally standardized scale. Using data from 634 teachers in 288 classrooms across 120 schools, our study indicated that approximately 21% of rural teachers were less than satisfied with their job, a number almost three times higher than other countries and regions across 52 countries reported by the same TIMSS & PIRLS survey (Figure 1). Moreover, our sample region is at the lowest rank of 52 countries or regions (Figure 2). Characteristics that were significantly correlated with teacher job dissatisfaction included being a male teacher, being a homeroom teacher, not having a management role in school, being a middle-aged teacher, and boarding school status. Several other variables were not correlated with levels of teacher job satisfaction, including certification status, credential status, number of degrees held by teachers, the school distance to the county seat, and school size.

Our study sought to measure the factors associated with low rates of teacher job satisfaction. In agreement with Sargent and Hannum [24], our data indicated that female teachers tend to be more satisfied as teachers. One study found that Chinese society has increasingly feminized teachers as a social group, possibly due to an ideological link between women’s domestic roles and their commitment to teaching [40,41]. As such, the social construction of masculinity and femininity could be linked to male teachers’ heightened dissatisfaction as teachers in rural China [40]. In addition, extant research indicates homeroom teachers work longer hours and have busier schedules [42]. An overwhelming workload has been associated with lower levels of teacher job satisfaction [22,24], which could account for the relatively low levels of satisfaction of homeroom teachers. Similarly, this could also explain the higher levels of satisfaction of teachers with management titles; they are not as overly burdened by heavy workloads or long hours. Finally, teachers at boarding schools had lower levels of job satisfaction. While teaching at a boarding school in rural China, teachers must take on more responsibilities due to the lack of professional auxiliary staff (especially since many rural schools have boarding facilities and large shares of students that live at school during the week), which, in turn, almost certainly has increased their workload and decreased their job satisfaction [43]. Additional research has also shown that rather than eliminating malnutrition, boarding schools in China may be exacerbating students’ malnutrition [44]. This negative correlation may lower teacher job satisfaction, as lower nutritional status seems to adversely impact school performance [45]. Similarly, the economic conditions of rural villages are often poor, and rural schools may reflect this reality [10,24]. 

Our data also indicate that several factors were not statistically associated with teacher job satisfaction. For example, teacher certification status and a teacher’s degree of education were not significantly correlated with teacher job satisfaction scores. It could be the case that teachers’ certification and education do not correlate with higher autonomy or pay. Especially in rural areas of China, certified teachers are difficult to recruit and retain; therefore, principals often hire substitute or temporary teachers who generally have low education levels and little to no formal teacher training [46]. Because most of their colleagues have lower levels of education, the teachers who have higher education and certification may feel that their additional qualifications are unnecessary.

In addition, school distance to the county seat was not significantly associated with teacher job satisfaction. Similarly, Sargent and Hannum found that school remoteness was not correlated with teacher job satisfaction, but that levels of community social resources, including community literacy and social support for schooling, were positively linked to teacher satisfaction [24]. As such, teacher satisfaction may be more closely linked to levels of perceived social support that could combat feelings of isolation often associated with remote living. Finally, we did not find a correlation between school size and teacher satisfaction. However, classroom size does not necessarily correlate with school size. Research indicates that one predictor of the stress level of teachers is their number of students, rather than the school population, which could account for this discrepancy [47]. As such, one potential area for further research includes a more in-depth analysis of correlates of teacher job dissatisfaction. In addition, a qualitative survey of teachers could better help shed light on correlates specific to rural China.

Limitations in our study include the use of cross-sectional data, which prevents researchers from drawing causal conclusions on the relation between the prevalence and correlates of teacher job satisfaction. In addition, this study relied on self-reporting questionnaires by teachers, which creates the potential for self-report biases (although other studies in the world that use data from these questionnaires are all subject to the same potential limitations). Moreover, when requesting permission for research, the local Education Bureau allowed us to survey math and Chinese teachers from fourth and fifth grade classrooms. Hence, due to the limited sample size and data availability, this is not a nationally representative sample, or representative of all schools and teachers in rural China. Finally, we are unable to rule out the possibility of omitted variable and selection biases, like teacher salary or general working conditions. Despite the scope of our data, we cannot account for all the potential characteristics that could affect teacher job satisfaction.

Despite these limitations, our study makes two crucial contributions to the literature. First, the most recent highly cited paper on teacher job satisfaction in rural China was published in 2005; this paper provides an updated estimate to the literature considering government efforts to improve the quality of rural schools. Second, the standardized measures used in this study help provide international context for levels of teacher job satisfaction in rural China.

## 5. Conclusions

With scores that place our sample of rural China in the lowest rank of 52 countries or regions, a high prevalence of teacher job dissatisfaction may pose an alarming obstacle to educating students elsewhere in rural China. The first step in addressing education equity issues like teacher job satisfaction is to assess and gauge their severity. While our study is preliminary, it is indeed one of the first in an underexplored research area that seeks to empirically assess what appears to be a critical issue. In doing so, our study provides substantial evidence of alarmingly low rates of teacher job satisfaction in our sampled area. While more data are necessary to better inform specific policy action, the evidence in the paper nonetheless supports the necessity of further research that could help policymakers understand how they can invest in the development of rural teachers so the education system in rural China can raise teacher job satisfaction and improve student outcomes.

## Figures and Tables

**Table 1:** Summary statistics of teacher characteristics.

Variable	Mean/Percentage	SD	Min	Max
Female, %	48.1%	0.50	0	1
Age (Year)	34.1	9.39	20	63
Age above 35 years, %	34%	0.48	0	1
Age above 50 years, %	7%	0.25	0	1
Certificated teacher, %	94.2%	0.23	0	1
Teaching experience, years	13.9	10.02	0	43
Teaching experience more than 5 years, %	80%	0.40	0	1
Teaching experience more than 20 years, %	23%	0.42	0	1
Home room teacher, %	60.5%	0.49	0	1
Principal, %	12.44%	0.33	0	1
Working hours per day (hours)	9.62	1.92	1	18
Less than 8 h	4.51			
8 h	24.88			
9 h	12.75			
10 h	38.1			
11 h and more	19.75			
Work over weekends	87.4			
Tutoring after school	35.93			
Teacher Education Degree, %				
Vocational HS	45.92			
Acd. HS	5.33			
Vocational College	42.32			
Acd. College	6.43			
Teacher rank, %				
No credentials	7.7			
Level 3	3.77			
Level 2	31.45			
Level 1	45.6			
Level High	11.48			

Number of teachers = 634.

**Table 2:** Summary statistics of school characteristics.

Variable	Mean	SD	Min	Max
Number of students	486.64	508.56	22	2144
Number of boarding students	47.64	107.95	0	532
Students per teacher	18.32	5.10	3.67	30.83
Distance from school to county seat, Km	30.43	14.96	2	70
Distance from school to farthest village, Km	6.73	5.55	1	30

Number of schools = 120.

**Table 3:** Teacher Job Satisfaction Scale.

Variable	Percent	SD
Raw Teacher Job Satisfaction (TJS) score (0–10)	4.66	3.15
Teacher Job Satisfaction category		
Less than Satisfied	20.68	0.41
Somewhat Satisfied	50.86	0.50
Very Satisfied	28.46	0.45
By items		
I am content with my profession as a teacher		
Never or almost never	45.77	0.50
Often	37.30	0.48
Very Often	16.93	0.38
I find my work full of meaning and purpose		
Never or almost never	21.63	0.41
Often	47.65	0.50
Very Often	30.72	0.46
I am enthusiastic about my job		
Never or almost never	21.63	0.41
Often	51.41	0.50
Very Often	26.96	0.44
My work inspires me		
Never or almost never	29.00	0.45
Often	47.81	0.50
Very Often	23.20	0.42
I am proud of the work I do		
Never or almost never	34.64	0.48
Often	40.75	0.49
Very Often	24.61	0.43

**Table 4:** Teacher and school characteristics and teacher job satisfaction.

	Raw Teacher Job Satisfaction Scores (0–10)	Difference
Gender		
Male	4.441	−0.533 **
Female	4.974	
Age **		
Below 25	5.025	
26–35	4.418	
36–45	4.602	
46+	5.236	
Homeroom Teacher		
No	5.016	0.586 **
Yes	4.429	
School Management Title		
No	4.483	−1.429 ***
Yes	5.912	
Certificated Teacher		
No	4.784	0.130
Yes	4.653	
School has boarding student		
No	4.758	0.278 *
Yes	4.480	
Teacher rank:		
No credentials	5.265	
Level 3	5.375	
Level 2	4.440	
Level 1	4.576	
Level High	5.205	
Teacher Education Degree		
1 = Vocational HS	4.464	
2 = Acd. HS	5.265	
3 = Vocational College	4.844	
4 = Acd. College	4.927	
Distance		
Less than 15 km	4.667	
16–30 km	4.973	
31–40 km	4.372	
41–50 km	4.417	
50 km+	4.525	
School Size (Sq. m)		
Samllest 25%	4.732	
50%	4.654	
75%	4.839	
Largest 25%	4.377	

Note: *** *p* < 0.01, ** *p* < 0.05, * *p* < 0.1.

## Data Availability

Data are available upon request.
